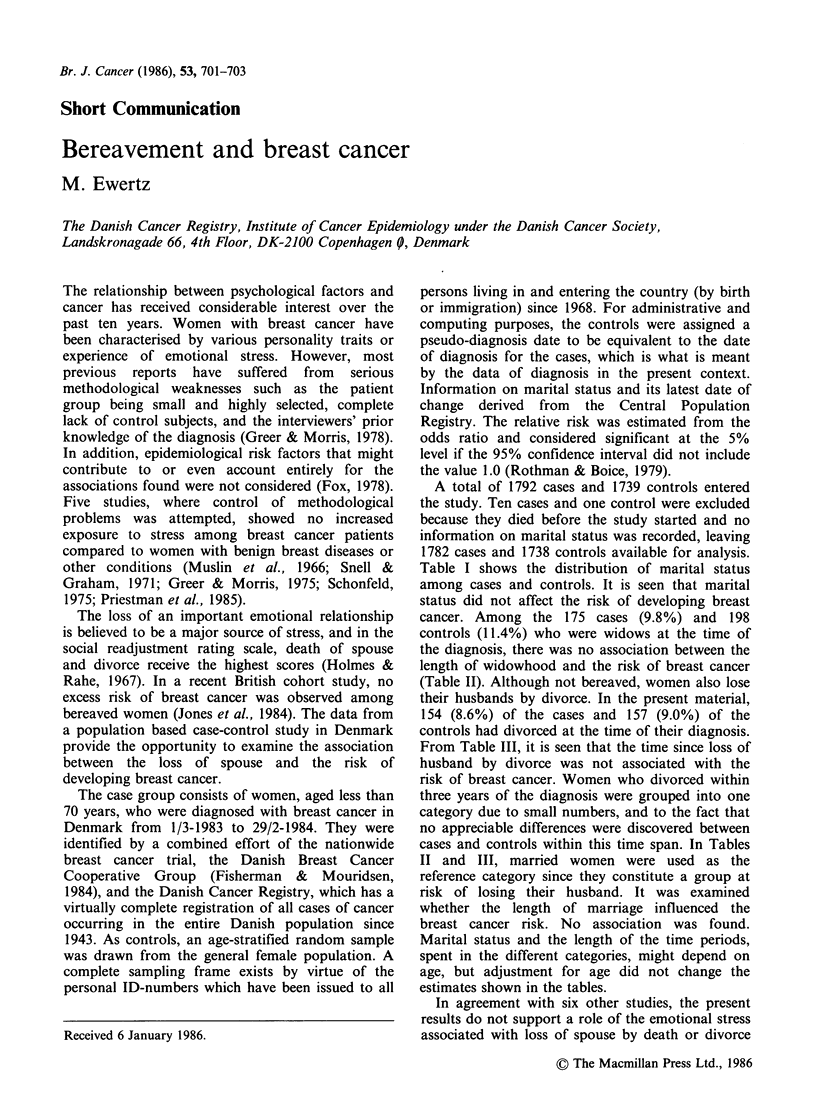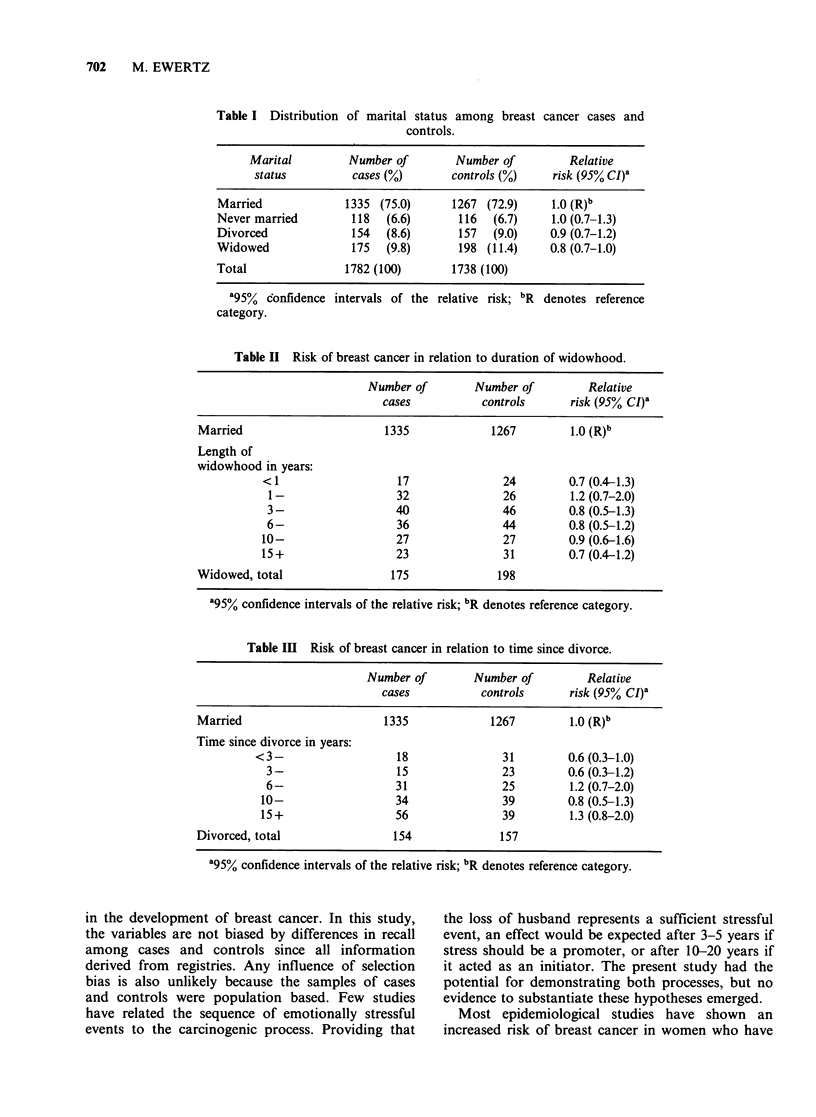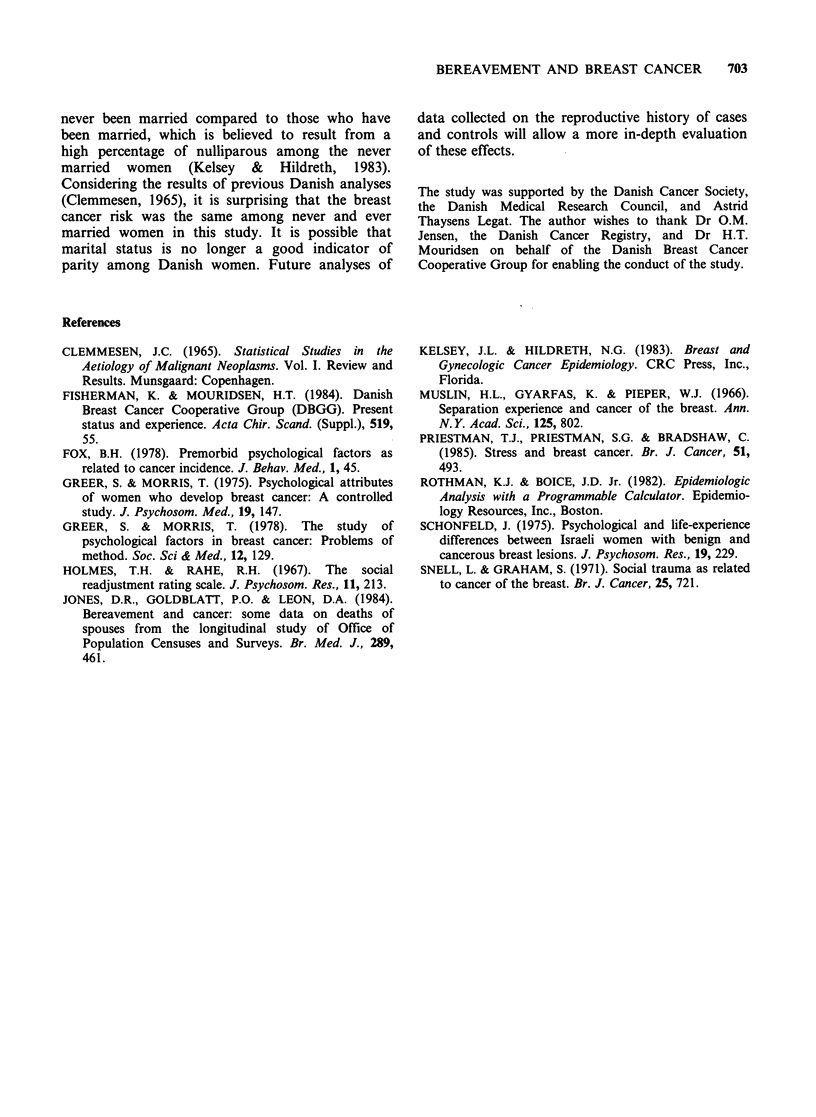# Bereavement and breast cancer.

**DOI:** 10.1038/bjc.1986.117

**Published:** 1986-05

**Authors:** M. Ewertz


*Br. J. Cancer* (1986), **53**, 701–703

### Short Communication

### Bereavement and breast cancer

M. Ewertz

*The Danish Cancer Registry, Institute of Cancer Epidemiology under the Danish Cancer Society, Landskronagade 66, 4th Floor, DK-2100 Copenhagen Ø, Denmark*

The relationship between psychological factors and cancer has received considerable interest over the past ten years. Women with breast cancer have been characterised by various personality traits or experience of emotional stress. However, most previous reports have suffered from serious methodological weaknesses such as the patient group being small and highly selected, complete lack of control subjects, and the interviewers' prior knowledge of the diagnosis (Greer & Morris, 1978). In addition, epidemiological risk factors that might contribute to or even account entirely for the associations found were not considered (Fox, 1978). Five studies, where control of methodological problems was attempted, showed no increased exposure to stress among breast cancer patients compared to women with benign breast diseases or other conditions (Muslin *et al.*, 1966; Snell & Graham, 1971; Greer & Morris, 1975; Schonfeld, 1975; Priestman *et al.*, 1985).

The loss of an important emotional relationship is believed to be a major source of stress, and in the social readjustment rating scale, death of spouse and divorce receive the highest scores (Holmes & Rahe, 1967). In a recent British cohort study, no excess risk of breast cancer was observed among bereaved women (Jones *et al.*, 1984). The data from a population based case-control study in Denmark provide the opportunity to examine the association between the loss of spouse and the risk of developing breast cancer.

The case group consists of women, aged less than 70 years, who were diagnosed with breast cancer in Denmark from 1/3-1983 to 29/2-1984. They were identified by a combined effort of the nationwide breast cancer trial, the Danish Breast Cancer Cooperative Group (Fisherman & Mouridsen, 1984), and the Danish Cancer Registry, which has a virtually complete registration of all cases of cancer occurring in the entire Danish population since 1943. As controls, an age-stratified random sample was drawn from the general female population. A complete sampling frame exists by virtue of the personal ID-numbers which have been issued to all persons living in and entering the country (by birth or immigration) since 1968. For administrative and computing purposes, the controls were assigned a pseudo-diagnosis date to be equivalent to the date of diagnosis for the cases, which is what is meant by the data of diagnosis in the present context. Information on marital status and its latest date of change derived from the Central Population Registry. The relative risk was estimated from the odds ratio and considered significant at the 5% level if the 95% confidence interval did not include the value 1.0 (Rothman & Boice, 1979).

A total of 1792 cases and 1739 controls entered the study. Ten cases and one control were excluded because they died before the study started and no information on marital status was recorded, leaving 1782 cases and 1738 controls available for analysis. Table I shows the distribution of marital status among cases and controls. It is seen that marital status did not affect the risk of developing breast cancer. Among the 175 cases (9.8%) and 198 controls (11.4%) who were widows at the time of the diagnosis, there was no association between the length of widowhood and the risk of breast cancer (Table II). Although not bereaved, women also lose their husbands by divorce. In the present material, 154 (8.6%) of the cases and 157 (9.0%) of the controls had divorced at the time of their diagnosis. From Table III, it is seen that the time since loss of husband by divorce was not associated with the risk of breast cancer. Women who divorced within three years of the diagnosis were grouped into one category due to small numbers, and to the fact that no appreciable differences were discovered between cases and controls within this time span. In Tables II and III, married women were used as the reference category since they constitute a group at risk of losing their husband. It was examined whether the length of marriage influenced the breast cancer risk. No association was found. Marital status and the length of the time periods, spent in the different categories, might depend on age, but adjustment for age did not change the estimates shown in the tables.

In agreement with six other studies, the present results do not support a role of the emotional stress associated with loss of spouse by death or divorce

Received 6 January 1986.

© The Macmillan Press Ltd., 1986

**Table I** Distribution of marital status among breast cancer cases and controls.

| *Marital status* | *Number of cases (%)* | *Number of controls (%)* | *Relative risk (95% CI)*[a] |
|---|---|---|---|
| Married | 1335 (75.0) | 1267 (72.9) | 1.0 (R)[b] |
| Never married | 118 (6.6) | 116 (6.7) | 1.0 (0.7–1.3) |
| Divorced | 154 (8.6) | 157 (9.0) | 0.9 (0.7–1.2) |
| Widowed | 175 (9.8) | 198 (11.4) | 0.8 (0.7–1.0) |
| Total | 1782 (100) | 1738 (100) | |

[a]95% confidence intervals of the relative risk; [b]R denotes reference category.

**Table II** Risk of breast cancer in relation to duration of widowhood.

| | *Number of cases* | *Number of controls* | *Relative risk (95% CI)*[a] |
|---|---|---|---|
| Married | 1335 | 1267 | 1.0 (R)[b] |
| Length of widowhood in years: | | | |
| <1 | 17 | 24 | 0.7 (0.4–1.3) |
| 1– | 32 | 26 | 1.2 (0.7–2.0) |
| 3– | 40 | 46 | 0.8 (0.5–1.3) |
| 6– | 36 | 44 | 0.8 (0.5–1.2) |
| 10– | 27 | 27 | 0.9 (0.6–1.6) |
| 15+ | 23 | 31 | 0.7 (0.4–1.2) |
| Widowed, total | 175 | 198 | |

[a]95% confidence intervals of the relative risk; [b]R denotes reference category.

**Table III** Risk of breast cancer in relation to time since divorce.

| | *Number of cases* | *Number of controls* | *Relative risk (95% CI)*[a] |
|---|---|---|---|
| Married | 1335 | 1267 | 1.0 (R)[b] |
| Time since divorce in years: | | | |
| <3– | 18 | 31 | 0.6 (0.3–1.0) |
| 3– | 15 | 23 | 0.6 (0.3–1.2) |
| 6– | 31 | 25 | 1.2 (0.7–2.0) |
| 10– | 34 | 39 | 0.8 (0.5–1.3) |
| 15+ | 56 | 39 | 1.3 (0.8–2.0) |
| Divorced, total | 154 | 157 | |

[a]95% confidence intervals of the relative risk; [b]R denotes reference category.

in the development of breast cancer. In this study, the variables are not biased by differences in recall among cases and controls since all information derived from registries. Any influence of selection bias is also unlikely because the samples of cases and controls were population based. Few studies have related the sequence of emotionally stressful events to the carcinogenic process. Providing that the loss of husband represents a sufficient stressful event, an effect would be expected after 3–5 years if stress should be a promoter, or after 10–20 years if it acted as an initiator. The present study had the potential for demonstrating both processes, but no evidence to substantiate these hypotheses emerged.

Most epidemiological studies have shown an increased risk of breast cancer in women who have never been married compared to those who have been married, which is believed to result from a high percentage of nulliparous among the never married women (Kelsey & Hildreth, 1983). Considering the results of previous Danish analyses (Clemmesen, 1965), it is surprising that the breast cancer risk was the same among never and ever married women in this study. It is possible that marital status is no longer a good indicator of parity among Danish women. Future analyses of data collected on the reproductive history of cases and controls will allow a more in-depth evaluation of these effects.

The study was supported by the Danish Cancer Society, the Danish Medical Research Council, and Astrid Thaysens Legat. The author wishes to thank Dr O.M. Jensen, the Danish Cancer Registry, and Dr H.T. Mouridsen on behalf of the Danish Breast Cancer Cooperative Group for enabling the conduct of the study.

### References

CLEMMESEN, J.C. (1965). *Statistical Studies in the Aetiology of Malignant Neoplasms*. Vol. I. Review and Results. Munsgaard: Copenhagen.

FISHERMAN, K. & MOURIDSEN, H.T. (1984). Danish Breast Cancer Cooperative Group (DBGG). Present status and experience. *Acta Chir. Scand.* (Suppl.), **519**, 55.

FOX, B.H. (1978). Premorbid psychological factors as related to cancer incidence. *J. Behav. Med.*, **1**, 45.

GREER, S. & MORRIS, T. (1975). Psychological attributes of women who develop breast cancer: A controlled study. *J. Psychosom. Med.*, **19**, 147.

GREER, S. & MORRIS, T. (1978). The study of psychological factors in breast cancer: Problems of method. *Soc. Sci & Med.*, **12**, 129.

HOLMES, T.H. & RAHE, R.H. (1967). The social readjustment rating scale. *J. Psychosom. Res.*, **11**, 213.

JONES, D.R., GOLDBLATT, P.O. & LEON, D.A. (1984). Bereavement and cancer: some data on deaths of spouses from the longitudinal study of Office of Population Censuses and Surveys. *Br. Med. J.*, **289**, 461.

KELSEY, J.L. & HILDRETH, N.G. (1983). *Breast and Gynecologic Cancer Epidemiology*. CRC Press, Inc., Florida.

MUSLIN, H.L., GYARFAS, K. & PIEPER, W.J. (1966). Separation experience and cancer of the breast. *Ann. N.Y. Acad. Sci.*, **125**, 802.

PRIESTMAN, T.J., PRIESTMAN, S.G. & BRADSHAW, C. (1985). Stress and breast cancer. *Br. J. Cancer*, **51**, 493.

ROTHMAN, K.J. & BOICE, J.D. Jr. (1982). *Epidemiologic Analysis with a Programmable Calculator*. Epidemiology Resources, Inc., Boston.

SCHONFELD, J. (1975). Psychological and life-experience differences between Israeli women with benign and cancerous breast lesions. *J. Psychosom. Res.*, **19**, 229.

SNELL, L. & GRAHAM, S. (1971). Social trauma as related to cancer of the breast. *Br. J. Cancer*, **25**, 721.